# Tobacco, Sunflower and High Biomass SRC Clones Show Potential for Trace Metal Phytoextraction on a Moderately Contaminated Field Site in Belgium

**DOI:** 10.3389/fpls.2018.01879

**Published:** 2018-12-21

**Authors:** Sofie Thijs, Nele Witters, Jolien Janssen, Ann Ruttens, Nele Weyens, Rolf Herzig, Michel Mench, Stijn Van Slycken, Erik Meers, Linda Meiresonne, Jaco Vangronsveld

**Affiliations:** ^1^Centre for Environmental Sciences University, Hasselt University, Diepenbeek, Belgium; ^2^Veterinary and Agrochemical Research Centre, CODA-CERVA, Tervuren, Belgium; ^3^Phytotech Foundation, Bern, Switzerland; ^4^BIOGECO, INRA, University of Bordeaux, Pessac, France; ^5^Laboratory of Analytical Chemistry and Applied Ecochemistry, Ghent University, Ghent, Belgium; ^6^Institute for Nature and Forest Research, Geraardsbergen, Belgium

**Keywords:** phytoextraction, metal, cadmium, zinc, short rotation coppice, sunflower, tobacco

## Abstract

Phytoextraction could be a potential management option for diffusely Cd-Zn-Pb-polluted agricultural land in Northeast Belgium. The use of high yielding crops with a sufficiently high metal accumulation is preferred as these are expected to both gradually decontaminate the soil while generating an income through biomass valorization. To find out which high biomass crop possessed the highest and most constant (in time) phytoextraction potential on these soils, different plant species and different mutants or clones of each species, were evaluated during consecutive years. Biomass production and metal accumulation of pre-selected tobacco somaclonal variants (*Nicotiana tabacum* L.) and pre-selected sunflower mutants (*Helianthus annuus* L.) were investigated for two productivity years, while the phytoextraction potential of experimental poplar (*Populus*) and willow (*Salix*) in short rotation coppice (SRC) was assessed at the end of the second cutting cycle (after two times four growing seasons). The tobacco clones and the sunflower mutants showed efficient extraction of, respectively, Cd and Zn, while the highest simultaneous extractions of Cd and Zn were gained with some SRC clones. Variation in biomass production and metal accumulation were high for all crops over the years. The highest biomass production was observed for the experimental poplar clone of the crossing type *Populus deltoides* (*P. maximowiczii x P. trichocarpa*) with 9.9 ton DW per ha per year. The remediation period to reach legal threshold values for the pseudo-total content of Cd in this specific soil was estimated to be at least 60 years. Combining estimated phytoextraction potential and economic and environmental aspects, the SRC option is proposed as the most suitable crop for implementing metal phytoextraction in the investigated area.

## Introduction

In the northeast of Belgium, an area of about 280 km^2^ is historically polluted by mainly cadmium (Cd), zinc (Zn) and lead (Pb) (Vangronsveld et al., 1995; Hogervorst et al., 2007). The negative impacts on inhabitants and the environment in general as well as economic losses in the agricultural sector urged regional policy makers to endorse remediation of the metal-polluted soils. Given the vastness of the area and the diffuseness, moderation and shallowness of the pollution, phytoextraction, using plants to extract metals from the soil and accumulate them in harvestable biomass, is proposed as a suitable remediation option (Ruttens et al., 2011). More specifically, cultivating non-food high biomass crops with moderate metal accumulation capacity is promising for this area as these crops result in both a gradual soil depollution by extracting metals as well as in an alternative income for the farmers.

Woody plants such as willow and poplar have been the topic of research over the past years for soil trace metal remediation (Mench et al., 2010; Courchesne et al., 2016). They are fast growing tree species often grown in short rotation coppice (SRC) for bioenergy production (Mola-Yudego et al., 2015). These species can also accumulate high concentrations of available metal(loid)s (Dickinson et al., 2009; Ruttens et al., 2011). In a number of European countries, phytoextraction of soil trace metals by willow and poplar was investigated during phytoremediation of metal-polluted soils, e.g., in Sweden (Perttu and Kowalik, 1997; Klang-Westin and Eriksson, 2003), Poland (Landberg and Greger, 1996; Perttu and Kowalik, 1997), France (Robinson et al., 2000), Denmark (Jensen et al., 2009), Switzerland (Hammer et al., 2003; Rosselli et al., 2003), the Czech Republic (Fischerová et al., 2006; Zárubová et al., 2015) and the United Kingdom (Dickinson and Pulford, 2005; French et al., 2006; Maxted et al., 2007). Although, overly the studies point to high variation in metal accumulation between the genera, and also at species and cultivar level, indicating that selection for metal accumulation and resistance traits is an important strategy to make differences in phytoextraction efficiency in the long term, though more data are needed to support this. Besides plant genetics, environmental factors such as soil pH, nutrient level, pollution concentrations, and soil microbiota differ widely between field sites, which makes a direct site-to-site comparison difficult. To account for these site-specific effects and to discriminate which are some of the most influential factors, knowledge can only be gained through more field studies.

In contrast to SRC, a fewer number of studies have looked into the potential of tobacco or sunflowers for metal extraction potential, though the studies that are there show promising results. For example, the capacity of *Nicotiana tabacum* L. to extract Cd from soil was reported in the early onsets of phytoextraction field experiments, by Mench et al. (1989). In this experiment soil Cd enrichment (5.4 mg Cd kg^-1^) invariably increased the Cd concentrations in plant parts, which varied from 10.1 to 164 mg kg^-1^ dry weight (DW). Moreover, they showed that between 75 and 81% of total Cd taken up by the plant was transported to the leaves, irrespective of the Cd level in the soil. Guadagnini (2000) also mentioned excellent Cd accumulation properties and a high biomass productivity for tobacco. Its metal extraction potential was further investigated at field scale in different countries (Vangronsveld et al., 2009; Fässler et al., 2010; Herzig et al., 2014). Sunflower (*Helianthus annuus* L.) is a bioenergy plant able to accumulate high amounts of several metals in its aerial tissues, gaining growing interest for phytoremediation purposes (De Maria and Rivelli, 2013; Kötschau et al., 2014). Phytoextraction of metal-polluted soils using sunflower was investigated before in pot trials (De Maria and Rivelli, 2013; Rivelli et al., 2014; Zalewska and Nogalska, 2014) and at field scale (Nehnevajova et al., 2007, 2009; Fässler et al., 2010; Herzig et al., 2014; Kötschau et al., 2014).

There still exist many reservations concerning the longer-term effectiveness of phytoextraction (Dickinson et al., 2009). Extrapolations of phytoremediation efficiency based on hydroponic and pot experiments are often unrealistic (Vangronsveld et al., 2009) and long lasting experiments at field scale are scarce (Dickinson et al., 2009). A large-scale field experiment in the polluted Campine region, in the Northeast of Belgium, dates back to 2006 (Ruttens et al., 2011; Van Slycken et al., 2013b, 2015) and offers a unique opportunity to investigate some aspects of this concern. Evaluation of high biomass crops on the metal-polluted field in Belgium was also part of the Greenland EU project (FP7-KBBE-266124)^1^.

This manuscript evaluates the phytoextraction potential of the above-mentioned high biomass crops based on longer-term field data, in the Campine region in Belgium. More specifically this paper addresses differences in biomass production and metal accumulation between pre-selected tobacco clones, sunflower mutants, and experimental poplar and willow clones, as well as variations throughout different years where possible. Annual crops (tobacco and sunflower) were cultivated for subsequent years, and results are reported over the years 2012–2014, while woody crops were examined after four growing seasons in 2011. Our data, despite the large variation in biomass and accumulation for all crops, show significant differences between plant genera, within mutants and clones, as well as over the years.

## Materials and Methods

### Site Description

**FIGURE 1:**
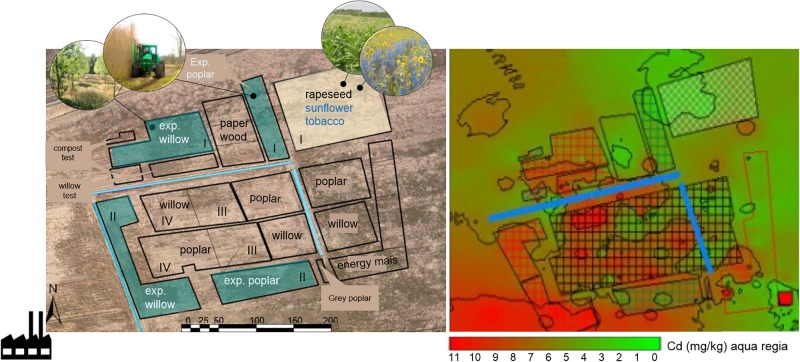
Scheme of the experimental field site in Lommel, and overview of the total Cd concentrations. The location of the sunflower and tobacco plot (2012–2014) analyzed in this study is highlighted in yellow (block I). The installed experimental willow and poplar plots (blocks I, II) are indicated in blue, the commercial willow and poplar plots were located in blocks III and IV (Van Slycken et al., 2015). Total Cd concentration map is based on an aqua regia destruction of subsurface (0–30 cm) collected soils (*n* = 47, year = 2006) (Van Slycken et al., 2015).

The Cd-Zn-Pb-polluted experimental field is located in Lommel, Belgium (51°12′41″ N; 5°14′32″ E). The site is a former maize field, taken out of production since 1999 and situated 500 m NE of a Zn smelter. The soil is a sandy soil (88% sand, 8% silt, 4% clay) with a pH of 4.6–5 (Meers et al., 2007b). The zinc smelter had its major environmental polluting production processes until the 1970s. During this period, metal slags were dumped in the soil around the factory and in the wider neighborhood. This study is part of a larger phytoremediaton experiment (10 ha) set up in 2006 as a collaboration between Hasselt University, Ghent University and the Research Institute for Nature and Forest (INBO) in Belgium (Van Slycken et al., 2013b). The field was subdivided into zones, and blocks for the different plantations, 2 ha for annual crops, and the remaining 4 ha was reserved for SRC (Figure 1).

Total metal concentrations throughout the field are very heterogeneous with hot spots of pollution dispersed across the site (Figure 1 and Table 1). The highest pollution concentrations are in the SW direction away from the former Zn smelter. The plantation plots are spread across the field (Figure 1).

**Table 1:** Average pH-H_2_O, total Cd, total Zn, and CaCl_2_ extractable Cd and Zn concentrations in the topsoil (0–30 cm) of the field site.

Block	pH-H_2_O	Aqua regia		CaCl_2_ extractable conc.	
			
		Cd mg kg^-1^	Zn mg kg^-1^	Cd mg kg^-1^	Zn mg kg^-1^
Low (I)	6.78 ± 0.13	4.05 ± 0.83	234.3 ± 37.9	0.21 ± 0.06	11.15 ± 5.12
Low (II)	6.3 ± 0.3	6.1 ± 2.2	344 ± 128	0.46 ± 0.18	3.9 ± 5.6
Mod (III)	6.5 ± 0.2	8.0 ± 1.6	445 ± 93	0.50 ± 0.12	2.2 ± 1.3
Mod-high (IV)	6.4 ± 0.2	8.4 ± 1.9	488 ± 97	0.59 ± 0.12	3.0 ± 2.5


*Values are mean ± SD of eight independent replicates.*

The sunflower and tobacco experimental field plot is located in the NE, block I (marked in yellow, Figure 1). The measured pseudo-total Cd concentrations ranged from 3.9 to 4.56 mg kg^-1^ DW soil and CaCl_2_ extractable Cd concentrations ranged from 0.2 to 0.34 mg kg^-1^ (Table 1). The cation exchange capacity (CEC) of this plot was on average 7.1 ± 0.7 meq per 100 g soil and the electrical conductivity (EC) was 54.24 ± 5.86 μS cm^-1^. Average Pb concentrations were 141.75 ± 16.3 mg kg^-1^ with CaCl_2_-extractable concentrations of 0.15 ± 0.04 mg kg^-1^. For Zn and Cd concentrations see Table 1. For this field plot, the pseudo-total Zn and Pb concentrations in the soil were lower than the remediation thresholds in Flanders (respectively, 282 and 200 mg kg^-1^ DW soil). The pseudo-total Cd-values exceeded the threshold value for an agricultural soil in Flanders (2 mg Cd kg^-1^).

The experimental willow and poplar clones were planted in 2006, and they were distributed over blocks I and II (Figure 1). Total Cd and Zn concentrations were higher for block II, closer to the Zn smelter than block I (Table 1). The pH-H_2_O for these areas is between 5.6 and 6.7, and pH-KCl of 5.5–6.3 (Ruttens et al., 2011).

### Climate Data and Field Maintenance

Climatological data for the cultivation period of tobacco and sunflower (June-July-August) for the years 2012 until 2014 are given in Supplementary Table S1. Compared to the normal values, i.e., mean climatological values for the 30-year period 1981–2010, some deviations were found for the year 2013. Relative air humidity, total rainfall and total days of rain were lower than normal (respectively, 7, 25 and 36% lower) while total hours of sunshine was higher (13%). Furthermore, the mean wind direction (NNE) was different from normal (SW) and less common in general in Belgium. For 2012 and 2014, differences compared to normal values were observed for total rainfall and total days of rain, which were higher than normal. The exceptional dry summer also had an effect on the plants, mainly the tobacco clones showed a significantly lower yield in 2013 and suffered the most from the drought, so we left this year out of the analyses.

### Field Plantation

#### Tobacco and Sunflower Set Up and Harvest

The plantations of tobacco and sunflower were started in 2011 and were followed up till 2014. Seeds of pre-selected *in vitro* bred tobacco clones (*N. tabacum* L. sp.) and mutant lines of sunflower (*H. annuus* L. sp.) were provided by Phytotech Foundation (PT-F) in Bern (Switzerland). Two tobacco somaclonal variant lines were tested: mother clone BAG (*Badischer Geudertheimer*) and its promising descendants NBCu104 and NBCu108 selected for higher metal accumulation and tolerance. The other tobacco line was the mother clone FOP (*Forchheim Pereg*) and derivatives NFCu715 and NFCu719. Second and third generation descendants of each of the selected clones were tested in subsequent years. Sunflower mutants belonged to three mutant line families (15-35-190-04, 86-35-190-04 and 14-185-04), all resulting of chemical mutagenesis for metal tolerance, of inbred line IBL04. From the fifth (M5) up to the eighth (M8) generation of different sunflower mutants were evaluated.

Seeds of the plants were germinated in the greenhouse under controlled conditions (day temperature 22°C, night temperature 18°C, air humidity 60%, photoperiod 15 h). This was done mainly to avoid the seed loss due to foraging rabbits or birds. Three week old seedlings were transferred to pots, acclimatized outside in the shade, before planting in the field.

Initially at the start of the field experiment, the soil was rototilled. For tobacco, a planting distance of 80 cm was chosen in 2012 (15,625 plants ha^-1^) while this was 60 cm in 2013 and 2014 (27,778 plants ha^-1^). 20–45 replicates were planted per clone. For sunflower, a planting distance of 25 cm in the row and 40 cm between the rows was followed (respectively, 166,667 plants ha^-1^ or 100,000 plants ha^-1^). 20–100 replicates per mutant were planted. In total the non-overlapping plots of sunflower and tobacco were 1,500 m^2^ each (Figure 1).

For maintenance, yearly, “Champions Blend” (Kooter B.V, Netherlands) was applied between the plans at a dose of 5 m^3^ per 100 m^2^ and mixed with the topsoil layer, to improve overall soil texture and water retention capacity. In 2014, the plot was treated with a glyphosate-based herbicide to remove all weeds and grasses.

Aboveground fresh weight (FW) production and height (H) of all tobacco and sunflower clones was determined on the field directly after harvest. A group of 10 plants per clone/mutant was chosen with a representative FW compared to the overall clone/mutant FW. All selected sunflower and tobacco plants were chipped individually using a garden chipper and chips were air-dried until constant weight (about 2 months). Thereafter, aboveground DW production was determined. DW production of the other, non-selected plants was estimated based on the regression equation expressing the FW-DW relationship of selected plants. In 2013, mass of produced sunflower seeds was also measured.

#### SRC Set Up and Harvest

In total 100 experimental poplar clones from 42 different families, and 160 experimental willow clones from 11 families were selected, produced by INBO, Belgium. The experimental poplar clones can be divided in three groups: *Populus trichocarpa* {T} clones, intraspecific crossings of *P. trichocarpa* × *P. trichocarpa* {T × T} and crossings of *P. trichocarpa* × *P. maximowiczii* {T × M} including two backcrossings to *P. deltoides* {D (T × M)}. The experimental willow clones can also be summarized in three groups: a *Salix alba* {A} group with purebred *S. alba* and intraspecific crossings of *S. alba* with *S. alba/S. rubens/S. fragilis*, a *S. viminalis* {V} group and a third group comprising crossings of *S. viminalis* × *S. viminalis* {V × V} derived from the second group. In April 2006, cuttings (20 cm) of all clones were planted on the experimental field in a twin row design with a row distance of 0.75 m between twins and 1.5 m between twin rows. For each tested experimental poplar and willow clone, 25–50 trees were planted in blocks in duplex repetition with planting distances of 90 (poplar) and 60 (willow) cm.

Plants were harvested in 2014 after a second 4-year growing season using the harvester “Stemster” from the Danish firm Nordic Biomass. It cuts the stems of twin rows close to the ground using two circular saws. For each clone biomass, 10 replicates were sampled and samples were collected from the stem, bark, and leaves. In the lab, stem and leaf tissue were separated and dried at 105°C to calculate DW of each of the fractions. In between the years also non-destructive plant biomass recordings were performed (Van Slycken et al., 2015). Because of the size of the harvesting machine, from each poplar and willow clone only one plot was harvested. Prior to harvest, height and diameter was determined of 20 representative plants per clone. The DW biomass data were used to calculate the biomass production at ha level, and expressed as productivity per tree according to Van Slycken et al. (2013b).

To collect samples for metal extractions, a mixed sample was used collected from six surrounding trees (mixture sample of ±200 g FW per clone), around each sampling location. In total, we chose five sampling locations for each clone dispersed over the field. The plants were defoliated, and divided into shoot, leaves and wood (stem, bark). On all plant fractions destructive analyses were performed to determine concentrations of Cd and Zn. Also, at the same moment soil samples were collected (0–25 cm) using a soil corer and analyzed for pH, and pseudo-total and CaCl_2_ extractable concentrations, as described above.

### Plant Metal Extractions and ICP-OES

All chipped and dried plant material was individually hammer-milled (Retsch SM100) to obtain a fine powder. To determine total Cd, Zn and Pb concentrations in the biomass, this powder was wet-digested in Pyrex tubes in a heating block. The digestion consisted of three cycles in 1 mL HNO_3_ (70%) and one cycle in 1 mL HCl (37%) at 120°C for 4 h. Samples were thereafter dissolved in HCl (37%) and diluted to a final volume of 5 mL (2% HCl) with Millipore water.

The extracts were subsequently analyzed with inductively coupled plasma optical emission spectrometry (ICP-OES, Agilent Technologies 700 Series). All samples were examined at least in triplicate. Blanks and certified reference material^®^ (trace elements in spinach, Standard Reference Material 1570a, National Institute of Standards and Technology, USA Department of Commerce) were included for quality control of the data. For pseudo-total metal concentrations, a reference soil (CRM 143 R Sewage Sludge Amended Soil, Community Bureau of Reference—BCR N° 230) was included for confirmation of the analysis.

### Soil Analyses

Soil samples were analyzed for physico-chemical parameters and total metals. Soil was oven-dried (60°C) and sieved (<2 mm). pH-H_2_O and pH-KCl were determined after 1 h of equilibration (120 rpm) with, respectively, deionized H_2_O and 1 M KCl in a 1:5 (w:v) solution. EC of the soil was determined using a conductivity meter (WTW LF340) and measured after 1 h of equilibration (120 rpm) with deionized H_2_O in a 1:5 (w:v) ratio. The effective CEC_e_ was calculated as the sum of cations (Ca/20+Mg/12+K/39+Al/9, cations in mg L^-1^) extracted by 1 M NH_4_Cl (Gillman and Sumpter, 1986). A 1:10 (w:v) extraction solution was shaken for 2 h (120 rpm) and cations present in the extract were measured using ICP-OES. Pseudo-total metal (Cd, Zn and Pb) concentrations of the soil samples was estimated by *aqua regia* digestion (Van Ranst et al., 1999). For this, 0.5 g of oven-dried soil was microwave digested in a HNO_3_-HCl solution (1:3 v:v) at 160°C (25 min ramp time, 10 min ventilation). CaCl_2_-extractable concentrations were determined in a 1:5 (w:v) extraction ratio as described previously (Van Ranst et al., 1999).

### Calculation of Metal Extraction Potential

The extraction potential was defined as the amount of metals removed from a soil and calculated based on the amount of metals accumulated in harvestable plant parts per unit of area and time. For tobacco and sunflower, the extraction potential was estimated by multiplying the mean above-ground DW production of the clone/mutant (kg ha^-1^ year^-1^) with the mean Cd, Zn and Pb concentrations in the evaluated plants (mg kg^-1^). For every poplar and willow clone, the extraction potential was calculated by multiplying yearly stem production with the concentration of metals in the woody biomass and expressed in g ha^-1^ year^-1^. As independent variables, also the effect of the block, crossing type, and Cd-concentrations in the soil was determined.

### Bioconcentration Factor and Hypothetical Remediation Time

For comparing phytoextraction efficiencies of tobacco and sunflower, bioconcentration factors (BCFs) were calculated. The BCF, defined as the ratio of metal concentration in above-ground biomass to (local) total soil metal content in the soil, allows to compare extraction efficiencies of crops even on different pollution levels. Results of the same clones/mutants (disregarding the generation) were averaged over the tested years to obtain more representative means.

For the best performing clones/mutants of the investigated species, hypothetical remediation periods were calculated to reduce pseudo-total Cd, Zn and Pb concentrations measured at the location of the tobacco and sunflower plots of 2012–2014, and in the middle of the field where poplar and willow was growing, respectively, referred to as moderate pollution levels, to pseudo-total remediation thresholds. For Cd, the time required to decrease the CaCl_2_-extractable (bioavailable) fraction to an assumed reference value for Flanders (based on values obtained for non-polluted soils) was also calculated.

### Statistical Analyses

Statistical analyses were performed in R 3.1.3 (R Development Core Team, 2013). The effect of tobacco clone/sunflower mutant and year on the biomass DW production per plant and Cd, Zn, and Pb concentrations in the biomass was analyzed using ANOVA. The QQ-plots were used to examine normality of the residuals. In case of non-normality, transformations of the outcome (logarithmic, inverse, square root, exponential) were performed. When an indication of non-normality was present for all these transformations, a Box-Cox was used. All decisions about the transformations of the outcomes were taken *a priori*. Model-robust SEs were used in all analyses due to potential differences in the variance of the outcome for different clones/mutants and years. Since interaction between clone/mutant and year was present in all analyses, the differences between clones/mutants for each year and the differences between years for each clone/mutant were analyzed separately. Two-by-two comparisons were conducted using Tukey correction for multiple testing.

## Results

### Tobacco: Biomass Production, Metal Accumulation and Extraction Potential

**FIGURE 2:**
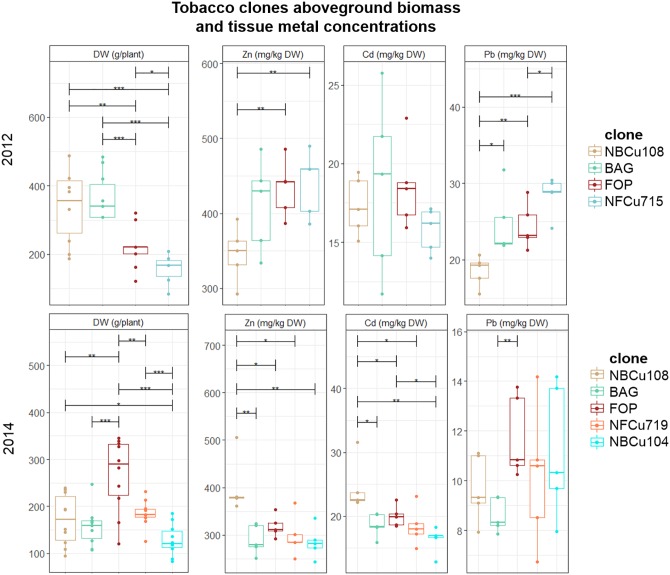
Mean aboveground DW production per plant (g) of *in vitro* bred tobacco clones tested in 2012 and 2014. Error bars are SEs (*n* = 10). Different colors represent the different clone, ranked according to highest mean above-ground biomass per year. BAG is the mother clone of NBCu clones while FOP is the mother clone of NFCu clones. Stars represent significant differences at the level of *p* < 0.05 (^∗^), *p* < 0.01 (^∗∗^) and *p* < 0.001 (^∗∗∗^) between clones within a year (ANOVA, Tukey-HSD). No significant differences were detected between the years for each clone (*t*-test).

Above-ground DW biomass production per plant differed significantly between the years (Figure 2). For example, mean above-ground DW production of a BAG plant varied from 150.68 ± 47.87 g in 2014 to 336.04 ± 75.13 g in 2012. Within 1 year, e.g., 2012, the highest mean above-ground biomass was observed for NBCu108 (328 ± 76) and its mother clone BAG (336 ± 75) which performed significantly better than FOP (238 ± 77) and its descendent clone NFCu715 (200 ± 75). In 2014, a different pattern was observed, with the highest biomass producing clone FOP, followed by NFCu719, and then the BAG clone with its derived clones NBCu108 and NBCu104.

When comparing metal concentrations per plants over the years, a variation in the best performing clones could be observed. For 2012, the plants with the highest metal concentrations of Zn and Pb were the clones with the lowest biomass. This is not so clear for 2014, as the highest biomass plant, FOP, also had the highest Pb concentration in the tissues. In 2014, NBCu108 had higher Zn and Cd concentrations in the aboveground tissues than its mother clone BAG, whereas this was not significant in 2012. FOP and its derived NFCu719 performed similar in Zn, Cd and Pb extraction in 2014. Only in 2012, NFCu715 extracted significantly more Pb than FOP. BAG and NBCu104 also performed similar in Zn, Cd and Pb extraction, not different from FOP and NFCu719 in 2014.

### Sunflower: Biomass Production, Metal Accumulation and Extraction Potential

Since rabbits consumed all sunflowers planted in 2012, no results were available for that year. Of all tested sunflower mutants grown, mean height varied between 106 cm (2014: 15-35-190-04 M8) and 147 cm (2013: 14-185-04 M5). Above-ground DW production per plant was similar in 2013 compared to 2014, but within each year statistical differences between the mutants could be observed. For example, mutant 15-35-190-M8 showed significantly lower production compared to 14-185-04-M5 and IBL04, but in 2014 they were not statistically different from each other. Here, 15-35-190-M8 showed higher productivity compared to 86-35-190-04-M8 and 15-35-190-04-M7. Across both years, the mutant showing the highest biomass was 14-185-04-M5 with on average 116 ± 51 mg kg^-1^ DW and 82.7 ± 33 mg kg^-1^ DW.

**FIGURE 3:**
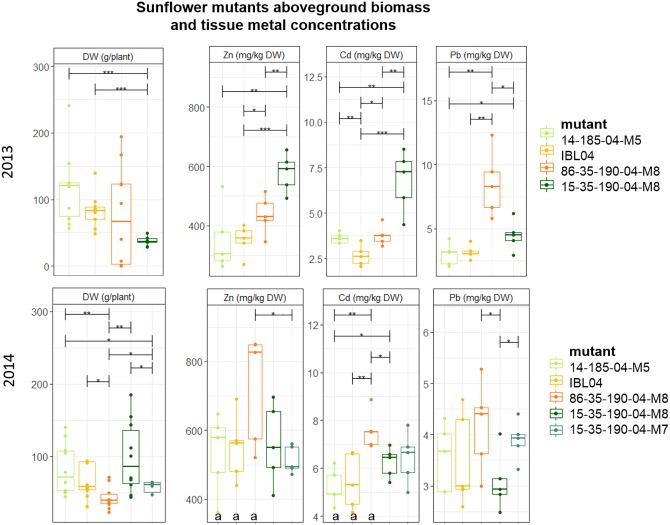
Mean aboveground DW production per plant (g) of sunflower mutants tested in 2013 and 2014. Error bars are SEs (*n* = 10). IBL04 is the mother line of all mutants. Stars represent significant differences at the level of *p* < 0.05 (^∗^), *p* < 0.01 (^∗∗^) and *p* < 0.001 (^∗∗∗^) between clones within a year (ANOVA, Tukey-HSD). Significant differences between the years for each clone are denoted with the letter a (*p* < 0.01, *t*-test).

Cadmium and Zn concentrations in above-ground biomass were significantly higher in 2014 compared to 2013 for the mother line IBL04, mutant 14-185-04 M5 and 86-35-190-04 M8 (Figure 3). For the mutant line family 15-35-190-04, similar shoot concentrations of Cd and Zn were found. For most of the mutants, shoot Pb concentrations did not differ considerably between 2013 and 2014. In 2013, the mutant 15-35-190-04 M8 showed significantly higher concentrations of Cd (6.76 ± 1.4) and Zn (578 ± 57) in above-ground biomass compared to IBL 04 (2.65 ± 0.5 mg kg^-1^ Cd and 350.9 ± 45.6 mg kg^-1^ Zn) and other derivatives. However, in 2014, this result was not repeated.

In addition to the metal concentrations measured in the above-ground leafy and stem tissue, we measured mean seed yield for the year 2013, next to Cd, Zn and Pb content in one mixed seed sample per mother line or mutant. In all cases, Pb values were below the detection limit. The average Cd and Zn concentrations measured ranged from 0.96 to 1.95 mg Cd kg^-1^, with the highest for 86-35-190-04 M8 (1.95) followed by 15-35-190-04 (1.42), IBL04 (1.34), and the lowest for 14-185-04 (0.96). Zn concentrations in seeds ranged from 64.28 to 111.16 mg Zn kg^-1^ with the highest Zn concentrations in IBL04, followed by 14-185-04 (87.25) and the other two mutants had on average 65.31 mg Zn kg^-1^.

### Metal BCF of Tobacco and Sunflower

The BCF of tobacco and sunflower was based on the local soil pseudo-total metal contents. From Table 2, it can be observed that for Cd and Zn, BCFs were almost always >1, while this was not the case for Pb. The BCF of Cd was the highest for tobacco, while the BCF of Zn was highest for sunflower. Overall, the BCF of Pb for tobacco and sunflower was very low (≤0.10).

**Table 2:** Mean BCF of Cd, Zn and Pb for tobacco clones and sunflower mutants.

Species	Clone/mutant	Cd (min-max)	Zn (min-max)	Pb (min-max)
Tobacco	BAG	2.8–6.3	1.1–2.1	0.06–0.2
	NBCu108	3.7–7.9	1.2–2.2	0.06–0.1
	FOP	3.9–4.5	1.2–1.8	0.07–0.2
	NBCu104	2.1–3.8	0.8–1.2	0.05–0.07
	NFCu719	3.6–5.7	1.06–1.5	0.04–0.2
Sunflower	IBL 04	0.5–0.9	1.14–2.3	0.01–0.03
	15-35-190-04	1.0–2.1	2.1–2.8	0.02–0.03
	86-35-190-04	0.9–1.9	1.5–2.4	0.04–0.08
	14-185-04	0.8–0.9	1.1–2.3	0.01–0.02


*BCF values are based on *in planta* metal concentrations expressed in ranges (min-max) for identical clones/mutants over two tested years, versus the mean pseudo-total metal concentrations in the soil.*

### SRC: Biomass Production, Metal Accumulation and Extraction Potential

**FIGURE 4:**
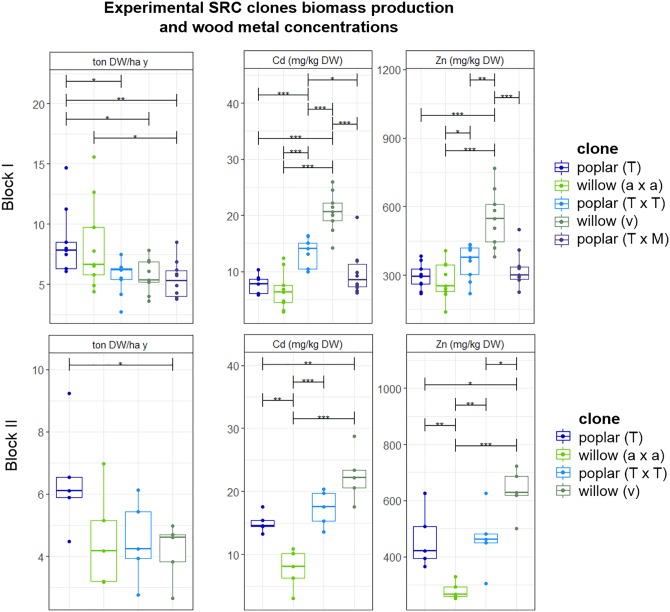
Mean DW stem biomass production per plant (ton) ha^-1^ year^-1^ and the metal concentrations in stem tissues of experimental willow and poplar clones after a second 4-year growth cycle, organized per block and crossing type. Error bars are SEs (*n* = 5–10). Stars represent significant differences at the level of *p* < 0.05 (^∗^), *p* < 0.01 (^∗∗^) and *p* < 0.001 (^∗∗∗^) between clones within a year (ANOVA, Tukey-HSD). No significant differences for each crossing type between the blocks were detected (*t*-test).

Biomass production and Cd and Zn concentrations in the stems of poplars and willows varied to a great extent from clone to clone and no clear distinction was observed between poplar and willow (Figure 4). Because of heterogeneity of Cd and Zn concentrations in the field, results were analyzed for the clones growing within one block, harvested in their fourth growth year. For block I, highest productivity was recorded for poplar crossing type *P. trichocarpa* (T) (6.3 ± 0.1 ton DW ha^-1^ year^-1^) and for intraspecific crossings of the *S. alba* group *S. alba/S. rubens/S. fragilis*, (a × a) with 6.2 ± 3.2 ton DW ha^-1^ year^-1^. In block II, poplar (T) showed the highest biomass productivity, not statistically different from block I. Though there was a trend in higher metal concentrations for all plants growing in block II compared to block I. For block I and II, the most promising metal extractor is the *S. viminalis* (v) willow group, with on average 20.6 ± 3.5 mg Cd kg^-1^ DW, and 548 ± 126 mg Zn kg^-1^ DW. The metal concentrations in *S. viminalis* (v) were almost two times higher compared to poplar clone T and willow (a × a), for both blocks. The second most promising clone is poplar (T × T), with on average 7.8 ± 3.2 mg Cd kg^-1^ DW, and 293 ± 57 mg Zn kg^-1^ DW in the stems.

Metal concentrations were analyzed in the leaves as well (data not shown). The highest leaf Zn cadmium concentrations were detected for *S. viminalis* × *S. viminalis* (v × v) with an average of 2,000 mg Zn kg^-1^ DW leaves, followed by *S. viminalis* (v) with an average of 1,600 mg Zn kg^-1^ DW, and *S. alba* 1,000 mg Zn kg^-1^ DW. Poplar clones T × M and T × T contained on average 1,600 mg Zn kg^-1^ DW leaves, and the Cd concentration was on average 35 mg kg^-1^ for both. Poplar (T) contained the lowest Zn (1,200) and Cd (17) concentrations in the leaves. Cd concentrations in the leaves were similar for willows compared to poplar, with on average 32 mg Cd for *S. viminalis* group (v) and (v × v), while *S. alba* group (a) contained 8 mg Cd.

### Biomass Productivity and Metal Extraction Efficiency of the Best Performing Tobacco, Sunflower and SRC Clones

**FIGURE 5:**
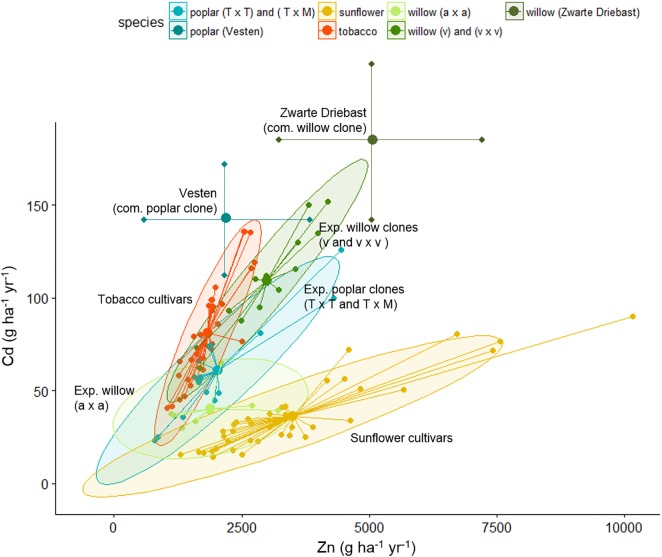
Cadmium and Zn extraction potential (g ha^-1^ year^-1^) of tobacco clones, sunflower mutants and poplar, willow clones evaluated on the experimental field. In case of tobacco and sunflower, extraction potential of identical clones/mutants were averaged over 2 years. Each clone is shown as a dot, connected with the spider-lines, and the overall average per group is indicated with the thick dot. Groups are colored, based on 90% ellipses. In case of commercial poplar and willow clones, results reflect data from Table 3.

Table 3 summarizes the biomass productivity, Cd and Zn extraction potentials of all evaluated species on the experimental field. The plants are organized per block, with the commercial SRCs growing in the blocks with highest pseudo-total metal concentrations (Van Slycken et al., 2013b, 2015). For each plant, the three best performing plants are shown. Figure 5 gives a graphical representation of the Zn and Cd extraction potential of all tested genera.

**Table 3:** Biomass production (ton DW ha^-1^ year^-1^), Cd and Zn extraction potential (g ha^-1^ year^-1^) of the three most promising tobacco clones, sunflower mutants, and experimental and commercial SRCs Tobacco and sunflower data were averaged over 2 years.

Block	Type	Biomass production (stem for SRC) (ton DW ha^-1^ y^-1^)		Cd extract. potential (g ha^-1^ year^-1^)		Zn extract. potential (kg ha^-1^ year^-1^)	
				
Species (#plants/ha)	Mutants, clones (# SRC clones)	Range (min max)	Mean (SD)	Range (min max)	Mean (SD)	Range (min max)	Mean (SD)
Tobacco I (15 625)	**NBCu108**	**4.3–5.2**	**4.8 (0.2)**	**67.8–143.8**	**105 (30)**	**1.9–2.6**	**1.9 (0.5)**
	FOP	3.07–5.0	4.9 (0.2)	66.1–194.8	95.2 (31)	1.2–2.7	1.7 (0.5)
	NFCu719	3.9–5.3	5.1 (0.7)	79.2–111.4	93.1 (10)	1.1–2.5	1.5 (0.3)
Sunflower I (27 778)	**15-35-190-04**	**5.6–7.1**	**6.3 (2.3)**	**31.5–76.2**	**42.5 (21)**	**1.9–7.5**	**5.6 (3.2)**
	14-185-04	5.7–10.1	8.6 (0.1)	26.5–80.3	35.6 (30)	1.7–6.7	4.2 (2.1)
	86-35-190-04	3.7–7.2	5.4 (2.4)	18.1–41.5	30.2 (2)	2.1–4.6	3.5 (2.1)
Poplar I (10 000)	**{D(T × M)} (1)**	**–**	**9.9**	**–**	**87.2**	**–**	**2.3**
	{T} (2)	6.2–6.4	6.3 (0.2)	61.5–105.1	83.3 (30)	2.5–2.7	2.6 (0.2)
	{T × T} (8)	6.1–14.7	8.5 (3.0)	35.5–125.9	67.4 (31)	1.3–4.4	1.5 (1.2)
Willow I (10 000)	**{v} (9)**	**3.6–7.8**	**5.7 (1.4)**	**83.3–152.0**	**115 (28)**	**2.9–4.1**	**3.1 (0.9)**
	{v × v} (1)	–	3.0	–	54.0	–	2.4
	{a × a} (14)	3.6–15.6	7.1 (3.5)	30.0–109	41.9 (23)	0.8–3.4	2.0 (1.2)
Poplar II (15 000)	**{T × M} (1)**	**–**	**5.1**	**–**	**99.0**	**–**	**1.5**
	{T} (3)	4.5–9.2	6.5 (2.4)	65.0–142.1	95.1 (41)	1.7–3.3	2.7 (0.3)
	{T × T} (15)	2.6–7.5	4.6 (1.6)	43.3–102.6	69.2 (22)	1.1–2.9	1.7 (0.4)
Willow II (15 000)	**{v × v} (5)**	**2.7–5.0**	**4.2 (0.9)**	**62–110**	**91.7 (18)**	**1.6–3.2**	**2.6 (0.6)**
	{a × a} (7)	3.2–7.0	4.5 (1.4)	16.1–56.7	29.9 (13)	1.1–3.2	1.1 (0.3)
Poplar III (10 000)	**Vesten (D × N)**	**–**	**6.2 (0.7)**	**–**	**143.5 (9)**	**–**	**2.2 (0.2)**
	Koster (D × N)	–	5.2 (1.4)	–	90.1 (41)	–	1.5 (0.6)
	GrimmingeD (T × D)	–	5.1 (0.1)	–	77.0 (7.1)	–	1.3 (0.1)
Willow III (15 000)	**Z. Driebast (Tr)**	**–**	**12.3 (3)**	**–**	**185 (49)**		**5.1 (0.5)**
	Loden (Da)	–	3.2 (0.7)	–	119 (18)		3.2 (0.8)
	Tora (S × V)	–	3.3 (0.4)	–	71.2 (14)		1.6 (0.2)


*D, *Populus deltoides*; M, *Populus maximowiczii*; N, *Populus nigra*; T, *Populus trichocarpa*; A, *Salix alba*; Da, *Salix dasyclados*; F, *Salix fragilis*; S, *Salix schwerinii*; Tr, *Salix triandra*; V, *Salix viminalis*; {}, collection of all experimental clones with this crossing type (# clones). Values are min-max and mean ± SD. “-” means data not available. Stem biomass production and Cd and Zn concentrations in the stems of commercial poplar clones were derived from Van Slycken et al. (2015), those of commercial willow clones from Van Slycken et al. (2013b). Extraction potentials of these commercial clones were calculated based on the abstracted data.*

*SRC data were collected from the first four growing seasons of most promising experimental clones and commercial clones planted on the field in 2006. The data from the commercial poplar clones were abstracted from Van Slycken et al. (2015), and the data from the commercial willow clones from Van Slycken et al. (2013b). The values in bold indicate the most promising clone within its genus.*

The tobacco clone NBCu-108 and sunflower mutant line 15-35-190-04 demonstrated highest average extraction of Cd and Zn in their groups. Sunflowers are the best Zn extractors, followed by some of the commercial willows. Poplar clones produced between 2.6 tons of stem biomass ha^-1^ year^-1^ [experimental clone *P. trichocarpa* × *trichocarpa* (T × T)] and 14.7 tons of stem biomass ha^-1^ year^-1^ [experimental clone (T × T)] while willow stem production varied between 2.7 (experimental clone v × v) and 15.6 [experimental clone of crossing type *S. alba* (a × a)] ton ha^-1^ year^-1^. Cadmium and Zn concentrations in stems ranged between, respectively, 35.5–142 g ha^-1^ year^-1^ and 1.1–4.4 kg Zn ha^-1^ year^-1^ for experimental poplars and between 30–152 and 1.1–4.1 kg^-1^ Zn ha^-1^ year^-1^ for willow. Highest average Cd and Zn extraction potentials within the experimental poplars were observed for a clone of the (T × M) and one of crossing type T (2.7 kg Zn ha^-1^ year^-1^). For willow, the commercial clone Zwarte Driebast showed the highest extraction potentials for Cd and Zn, respectively, 185 g Cd ha^-1^ year^-1^ and 5.1 kg ha^-1^ year^-1^.

When comparing the evaluated species, sunflower mutants clustered together at high Zn concentrations and low Cd extraction concentrations (Figure 5). The tobacco clones exhibited rather low Zn extraction and showed moderate amounts of Cd removal. The experimental poplar and willow clones covered a large range of Cd and Zn extraction and indicated a rather linear trend in combined Cd and Zn removal. While poplar crossings seemed to cluster in one group with a broad range of Cd extraction, willow experimental clones fell into two groups, the *S. alba* group *S. alba/S. rubens/S. fragilis*, (a × a) and the *S. viminalis* (v) and *S. viminalis x S. viminalis* (v × v). Between these groups, willow v showed the highest potential for Cd extraction, while the *S. alba* group in contrary had very low Cd extraction potential and a low to moderate Zn extraction. The best commercial willow clone (Zwarte Driebast) and most promising commercial poplar crossing type (Vesten) were plotted on top (mean ± SD, abstracted from Van Slycken et al., 2013b, 2015). Taking into account that these commercial clones were growing in soils with higher total metal concentrations (block III and IV), the data suggest that Zwarte Driebast holds most promise for Cd extraction and to some extend Zn, and performs better than the experimental clones. Vesten is also a good Cd extractor but less than Zwarte Driebast, and much less for Zn. Calculated extraction potentials ranged between 18 and 80 g Cd and 1.7 and 7.5 kg Zn ha^-1^ year^-1^ (Table 3). Over the 2 years, the three best performing sunflower mutants were 15-35-190-04 M7, 14-185-04 and 86-35-190-04 M8. Their average biomass productivity ranged from 3.7 to 10.1 ton DW ha^-1^ year^-1^.

### Calculated Hypothetical Remediation Times

Calculated hypothetical remediation times for these best performing clones/mutants revealed that the shortest depollution periods for Cd as well as Zn concentrations on the experimental field would most likely be obtained with Zwarte Driebast (Table 4). It would last a time span of 60 ± 36 years to decrease pseudo-total concentrations of the moderately polluted soil to pseudo-total remediation thresholds. When calculating the remediation time using CaCl_2_-extractable Cd concentrations, much shorter time spans (e.g., 5 ± 3 years for Zwarte Driebast) were estimated to reach CaCl_2_-exchangeable Cd concentrations that were found in unpolluted soils in Flanders (Meers et al., 2007a). Phytoextraction of Pb from the polluted soil revealed to be highly unrealistic when using the tested species/clones/mutants.

**Table 4:** Hypothetical remediation times (years) of best performing clones/mutant of tested species to reduce 1 mg of Cd, Zn and Pb kg^-1^ DW soil for high or moderate field Cd, Zn and Pb concentrations to remediation thresholds (2 mg Cd, 282 mg Zn and 200 mg Pb kg^-1^ DW soil).

Hypothetical remediation times (years)		Contamination level:			
	
		High			Moderate
		
Species	Clone/mutant	7 mg Cd kg^-1^ DW	429 mg Zn kg^-1^ DW	217 mg Pb kg^-1^ DW	4 mg Cd kg^-1^ DW
Poplar	D × (T × M)	336	320	n.d.	134
Willow	Zwarte Driebast	150 ± 90	181 ± 107	n.d.	60 ± 36
Tobacco	NBCu-10-8	401 ± 211	657 ± 345	1,864 ± 1,145	160 ± 84
Sunflower	15-35-190-04	744 ± 407	250 ± 152	4,250 ± 2,380	298 ± 163


*Assumptions made were: (i) species’ extraction potentials are independent of soil metal concentrations, (ii) total soil metal content decreases linearly due to a constant yearly extraction, (iii) contamination and rooting depth are 0.5 m, and (iv) soil density is 1,250 kg m^-3^.*

In Flanders, remediation criteria are site-specific as they are a function of destination type, clay, organic matter content and pH (Vlarebo, 2008). For the area under investigation, calculated pseudo-total remediation threshold values for soil were 2 mg Cd, 282 mg Zn and 200 mg Pb kg^-1^ DW soil.

## Discussion

### Biomass Production Tobacco Sunflower and SRC

Tobacco and sunflowers plant height and DW production per plant differed significantly among the years. Since both crops were grown next to each other on the same plots, this might suggest that they both are quite susceptible to yearly variations in climatological conditions, field preparation and maintenance actions and/or that the quality of the seeds might differ substantially between years. For example, the abnormal low tobacco biomass production in 2013 (data not shown), could be related to (a combination of) three factors: (1) plantlets were quite old (about 11 weeks) when the field was ready for planting. A growth spurt might already have taken place when the plants were still in pots, hampering their biomass production; (2) because no weed control was performed before and after planting, a considerable amount of weeds (mainly *Polygonum* sp.) between the tobacco plants was competing for nutrients and water; (3) in comparison with the other years and normal values, the quantity of rain was lower and there was more sun which might have caused drought stress to some extent. Above-ground yields of tobacco not only varied considerably over the years (Figure 2) but also did not reach yield values like reported by Kayser et al. (2000) (10–12.5 t ha^-1^ year^-1^), Fässler et al. (2010) (8.5–10.5 t ha^-1^ year^-1^) and Herzig et al. (2014) (24.7–37.5 t ha^-1^ year^-1^) obtained in phytoremediation field experiments in Switzerland.

Above-ground production of an IBL 04 sunflower plant on the metal-polluted soil in Lommel was similar to biomass productions of this inbred line found on the metal-polluted site in Rafz, Switzerland (93.7, 68 ± 17 and 78 ± 8.6 g) (Nehnevajova et al., 2007, 2009). Above-ground yields per hectare and year are rather low in comparison with above-ground yields reported for sunflowers cultivated on other metal-polluted soils in Switzerland, ranging from 7.5 up to 29 t ha^-1^ year^-1^ (Kayser et al., 2000; Fässler et al., 2010; Herzig et al., 2014) (Figure 3 and Table 3).

After four growing seasons of poplar and willow clones on the field in Lommel, biomass productivity differed considerably between clones but was in general quite low (mostly <6 t ha^-1^ year^-1^; Table 3). Biomass productivity levels of SRC depend on site-specific conditions, clonal selection, climatic conditions, plant spacing and management. For willow SRC, expected biomass productivity is between 6 and 10 t ha^-1^ year^-1^ in Sweden (Dimitriou et al., 2006) while higher values (10–20 t ha^-1^ year^-1^) were considered common by Maxted et al. (2007). Annual yields reported for poplars in SRC are between 10 and 15 t ha^-1^ in less intensive conditions (Laureysens et al., 2004a). Zegada-Lizarazu et al. (2010) mentioned an average biomass yield between 10 and 12 t ha^-1^ year^-1^ for poplar and willow in temperate climates. The lower productivity levels on the Lommel field site can be attributed to the nutrient poor, sandy characteristics of the soil (Van Slycken et al., 2015). Given the absence of fertilization and irrigation, the productivity of willow and poplar clones can be expected to be less in comparison with SRC cultures on more fertile soils. Furthermore, yields obtained after the first growing seasons tend to be lower than yields from later cutting cycles (Aronsson et al., 2014; Van Slycken et al., 2015) since, during the 1st years, a plant will allocate a considerable amount of its energy for the establishment of its root system. Also for Cd and Zn concentrations in the stem, obvious differences exist between clones (Table 3). Differences in metal uptake between cultivars were also reported by Landberg and Greger (1996, 2002), Granel et al. (2002); Mleczek et al. (2010), Ruttens et al. (2011) and Van Slycken et al. (2013b) (for willow) and Laureysens et al. (2004b) (for poplar). The high variability in stem biomass production and stem Cd and Zn concentrations for a commercial clone can partly be attributed to the heterogeneity of the field given that the same clone is planted (and measured) in different plots on different locations of the field. In case of the experimental groups, the large ranges are due to clonal differences as explained above.

### Metal Phytoextraction Potential of Tobacco, Sunflower and SRC

The significant differences in concentrations of Cd, Zn and Pb for tobacco clones or sunflower mutants throughout the years cannot be due to differences in soil metal content since all experiments were conducted in a restricted part of the field with similar soil characteristics (Figure 1 and Table 2). Also Fässler et al. (2010) reported considerable year-to-year variations in metal accumulation of tobacco and sunflower in field trials. It is speculative which (combination of) factors (climate conditions, seed quality/generation, field preparation and management, planting distance…) account for these differences between years.

The tobacco and sunflower cultivation at the Lommel field provided some information concerning potentially stable improvements after selection based on somaclonal variation and conventional *in vitro* breeding of tobacco and chemical mutagenesis of sunflower. Over the years, mean Cd and Zn removals of tobacco clone NBCu-10-8 were higher than mean values of BAG (Figure 2) which might indicate an improved tobacco clone for metal phytoextraction, although more research is required for confirmation. Regarding the sunflowers, averaging extraction potentials over the years 2013 and 2014 revealed a slight extraction improvement for mutant lines 15-35-190-04 and 14-185-04 in comparison with the IBL 04 control for Cd (Figure 3). The large biomass increments of these mutants compared to control, the “giant mutants” reported by Nehnevajova et al. (2007, 2009), were not observed in this case. The vegetative propagation of selected clones of willow and poplar is an important advantage to this concern. Vegetative propagation helps to maintain the improved characteristics of a certain genotype/cultivar/clone/variant (Zegada-Lizarazu et al., 2010). Furthermore, using stem cuttings to establish clonal plantations is expected to reduce variability between plants compared to plants raised from seeds (Dickinson et al., 2009).

Variability of extraction potentials of all evaluated clones/mutants is high (Figure 4), as a result of the heterogeneity of the field (for the SRC clones) and yearly variations in many factors (for tobacco and sunflower). In general, however, extraction potentials (Figure 2) together with BCFs (Table 4) suggest sunflower as a highly efficient Zn extractor and tobacco as a more prominent Cd extractor, confirming previous findings (Kayser et al., 2000; Fässler et al., 2010). BCFs ≥1 furthermore confirm efficient extraction (accumulation of metals in the crops relative to the soil) (Dickinson and Pulford, 2005; Kötschau et al., 2014) of Cd and Zn by sunflowers, and tobacco. Most evaluated poplar and willow clones showed phytoextraction potentials between that of tobacco and sunflower. However, the large range of (combined) Cd and Zn extraction covered by commercial willow clones and group means of experimental poplars and willows lead to optimism concerning clone selection (construing experimental groups) and/or conventional breeding approaches that may provide clones with a higher combined extraction of Cd and Zn. Furthermore, high combined metal extraction potentials for the commercial willow clone Zwarte Driebast indicates that this clone possess high efficiencies regarding Cd and Zn extraction in comparison to tobacco and sunflower (Figure 5). In addition, harvesting the leaves of SRC trees (which was performed in this research but not the main focus) and yield increments expected with increased age of the SRC plantations (see above) might even considerably increase metal extraction potentials of SRC clones (in later cutting cycles).

Phytoextraction of Pb using the species tested in Lommel is utopia (Figures 2, 3). The BCFs indicate a generally very low translocation of Pb to the above-ground biomass. Moreover, its low bioavailability in the soil, and even increased inactivation by a vegetation cover (Chaney et al., 1997), makes that soil Pb concentrations, even when exceeding remediation thresholds, rarely cause problems for agriculture (plant Pb uptake from soil) or the environment in general (spreading risks).

Caution needs to be taken with the calculated remediation times (based on pseudo-total or CaCl_2_-extractable fractions) (Table 4). Firstly, all tested plant species are assumed to possess a steady extraction potential, independent of soil metal concentrations. However, it was not evaluated to which extent extraction potentials of sunflower, tobacco, willow or poplar depend on soil pollution levels. Secondly, the yearly linear decrease in total soil metal concentration due to phytoextraction most likely is a simplistic approximation compared to the real situation. It assumes that a species’ biomass production, its metal accumulation (or at least the product of both) and the bioavailability of metals in the soil does not change over time. Several authors (Robinson et al., 2003, 2006; Koopmans et al., 2007; Van Nevel et al., 2007; Manzoni et al., 2011) proposed decay models incorporating, to some extent, soil chemistry (with all kinds of sorption, retention and leaching processes to describe evolutions in the “bioavailable” metal pool), changes in plant metal accumulation and biomass production over time. However, involving more factors increases uncertainty and since no model is acknowledged to be valid in all cases, the simplest approach is used here. The calculated remediation times of the investigated crops differ enough to conclude that willow clone Zwarte Driebast would need the shortest time to decrease the metal contents in this specific soil to the legislative threshold/adopted reference value(s). Furthermore, a more profound study of the individual clones in the experimental INBO crossing types might unravel other clones suitable for phytoextraction purposes. The tobacco clones and sunflower mutants used resulted from, respectively, *in vitro* breeding and mutagenesis followed by continuous breeding and selection for improved phytoextraction efficiencies. Therefore, further enhancement of the remediation potential in these groups is not very likely.

Whether to rely on remediation times based on pseudo-total (“total”) or CaCl_2_-exchangeable (“bioavailable”) metal concentrations is disputable. In many countries “total” metal concentrations are used in legislation. However, very promising prognosis of short depollution times of only a few years, can be made for the phytoextraction of the “bioavailable” metal pollution in soils that is adopted in, e.g., Switzerland (Vangronsveld et al., 2009; Herzig et al., 2014). This “bioavailable” pool of metals in soil is justifiably regarded as the main risk for pollution of both, food chains and groundwater (Karlaganis, 2001). Application of the “bioavailability” concept in risk assessment and management of polluted sites is increasing (Onwubuya et al., 2009; Kumpiene et al., 2014), considering that the risks for human health and ecosystems in metal-polluted soils are often poorly predicted by the total metal concentrations (McLaughlin et al., 2000). Several authors described a replenishment of the “bioavailable” metal pool (Zhang et al., 1998; Whiting et al., 2001; Hammer and Keller, 2002; Keller and Hammer, 2004; Fischerová et al., 2006), whereas Herzig et al. (2014) showed the relative stability of labile Zn topsoil concentrations (NaNO_3_-extracts) 1–3 years after stopping a 5 years phytoextraction treatment in Switzerland. In any case, this major consideration definitely demands for further investigation as also mentioned by Van Nevel et al. (2007). In addition, the requirements and the protocols of assessing “bioavailability” still differ considerably between European countries. Moreover, in Flanders no “bioavailable” remediation thresholds are acknowledged. Therefore, in this study, further considerations were based on remediation times calculated using pseudo-total metal concentrations.

These estimated remediation periods are long and generally considered too long for the implementation of phytoextraction as a stand-alone remediation technology [e.g., Blaylock and Huang (2000) suggested a period of about 10 years as threshold to render the technology economically feasible in itself]. Therefore, this research (as well as almost all evaluations of this matter in literature) emphasizes the necessity to combine phytoextraction with other opportunities. Synergies between social, economic as well as environmental agendas seem indispensible for the justification, advancement and eventual implementation of metal phytoextraction (Dickinson et al., 2009). Firstly, phytoextraction crops may generate economic revenues, for example, through conversion of produced biomass. Secondly, the growth of high biomass crops may restore ecosystem services (e.g., CO_2_ abatement, improving quality of soil, water, air…). Finally, social benefits (recreation, educational value, visual and aesthetical power) might arise from “green” remediation technologies and public acceptance is considered high (Kennen and Kirkwood, 2015). These external and indirect advantages of growing phytoextraction crops might not only compensate for long remediation times but, together with the remediation potentials, also determine the overall sustainability and effectiveness of a metal phytoextraction treatment. The way of thinking about metal phytoextraction as a larger concept of sustainable and risk mitigating land use is illustrated in this manuscript by means of a case study in Belgium.

Given the extraction potentials of tobacco, sunflower and SRC of willow and poplar in a case study in Belgium and available information on economics (biomass conversion) and environmental benefits of cultivating these crops so far, it is concluded that SRC would be the most interesting crop for metal phytoextraction in the investigated area. Besides this, it was an interesting finding that sunflower mutants are more suited for Zn extraction while tobacco plants are the better choice for Cd, and some of the most performing new mutants are identified for further studies. Finally, the optimal combination of the properties of a clone with high metal uptake capacities combined with high biomass productivity could lead to the formation of groups of clones showing high potential for trace metal phytoextraction. These results can then be incorporated into future breeding programs, research and rotation coppices.

In future, more elaborate investigations should also be dedicated to optimizing metal-enriched crop conversion in order to become sustainable and economically profitable. For the conversion of biomass in this case study, a prominent role seems to be reserved for pyrolysis, and the generation of metal-enriched activated carbon for filter medium purposes especially deserves further attention. Furthermore, finding a way to reward for the restoration of ecosystem services (including CO_2_ abatement) when growing phytoextraction crops will be crucial for the eventual implementation. In addition, also benefits related to social topics (e.g., recreation, education, design…) should be recognized and compensated in some way. Finally, conflicts between the factors determining the overall sustainability of a phytoextraction plantation might arise and case-to-case evaluations impose themselves to obtain intelligently designed phytoextraction concepts.

## Conclusion

The shortest estimated remediation time for simultaneous Cd and Zn clean-up was obtained with the commercial willow clone Zwarte Driebast. The best tobacco clone identified in this study for Cd phytoextraction was NBCu-108, and the most promising sunflower mutant line for Zn extraction was 15-35-190-04. The experimental willow and poplar clones show a large range of combined Cd and Zn extraction providing a substantial basis for optimism that clone selection and/or conventional breeding approaches may produce additional clones with high combined extraction of Cd and Zn.

A drawback still of metal phytoextraction using the evaluated high biomass crops, is the long period of time (>60 years) that would be needed to decrease “total” metal concentrations in the soil to legal threshold values. Although much shorter times are estimated when adopting “bioavailable” metal concentrations, these outcomes are still not generally accepted due to the uncertainty regarding equilibria between the various metal species in the soil and the eventual replenishment of the “bioavailable” metal pool on the longer term. Economic revenues through biomass conversion and a rewarding for environmental benefits of a phytoextraction crop plantation are crucial for large-scale, commercial implementation of metal phytoextraction.

## Author Contributions

JV, NWe, NWi, and AR conceived the study design. JV, NWe, and NWi coordinated the execution of the project by JJ and ST. ST and NWi wrote this manuscript in collaboration with JV and NWe. JV is promoter of the study. All authors contributed to the elaboration of the study design and took part in reviewing the methods, each member contributed specifically to the parts of the study corresponding with their own expertise. All authors read and approved the final version of the manuscript.

## Conflict of Interest Statement

The authors declare that the research was conducted in the absence of any commercial or financial relationships that could be construed as a potential conflict of interest.

## Footnotes

1 http://www.greenland-project.eu/

## References

[B1] Aronsson P. Rosenqvist H. Dimitriou I. (2014). Impact of nitrogen fertilization to short-rotation willow coppice plantations grown in Sweden on yield and economy. *BioEnergy Res.* 7 993–1001. 10.1007/s12155-014-9435-7

[B2] Blaylock M. J. Huang J. W. (2000). “Phytoextraction of metals,” in *Phytoremediation of Toxic Metals: Using Plants to Clean Up the Environment*, eds Raskin I. Ensley B. D. (Hoboken, NJ: Wiley), 53–70.

[B3] Chaney R. L. Malik M. Li Y. M. Brown S. L. Brewer E. P. Angle J. S. (1997). Phytoremediation of soil metals. *Curr. Opin. Biotechnol.* 8 279–284. 10.1016/S0958-1669(97)80004-3 9206007

[B4] Courchesne F. Turmel M.-C. Cloutier-Hurteau B. Constantineau S. Munro L. Labrecque M. (2016). Phytoextraction of soil trace elements by willow during a phytoremediation trial in Southern Québec, Canada. *Int. J. Phytoremediat.* 19 545–554. 10.1080/15226514.2016.1267700 27996300

[B5] De Maria S. Rivelli A. R. (2013). Trace element accumulation and distribution in sunflower plants at the stages of flower bud and maturity. *Ital. J. Agron.* 8:9 10.4081/ija.2013.e9

[B6] Dickinson N. M. Pulford I. D. (2005). Cadmium phytoextraction using short-rotation coppice *Salix*: the evidence trail. *Environ. Int.* 31 609–613. 10.1016/j.envint.2004.10.013 15788201

[B7] Dickinson N. M. Baker A. J. Doronila A. Laidlaw S. Reeves R. D. (2009). Phytoremediation of inorganics: realism and synergies. *Int. J. Phytoremediat.* 11 97–114. 10.1080/15226510802378368 28133994

[B8] Dimitriou I. Aronsson P. Weih M. (2006). Stress tolerance of five willow clones after irrigation with different amounts of landfill leachate. *Bioresour. Technol.* 97 150–157. 10.1016/j.biortech.2005.02.004 16154512

[B9] Fässler E. Robinson B. H. Stauffer W. Gupta S. K. Papritz A. Schulin R. (2010). Phytomanagement of metal-contaminated agricultural land using sunflower, maize and tobacco. *Agric. Ecosyst. Environ.* 136 49–58. 10.1016/j.agee.2009.11.007

[B10] Fischerová Z. Tlustoš P. Száková J. Šichorová K. (2006). A comparison of phytoremediation capability of selected plant species for given trace elements. *Environ. Pollut.* 144 93–100. 10.1016/j.envpol.2006.01.005 16516363

[B11] French C. J. Dickinson N. M. Putwain P. D. (2006). Woody biomass phytoremediation of contaminated brownfield land. *Environ. Pollut.* 141 387–395. 10.1016/j.envpol.2005.08.065 16271426

[B12] Gillman G. Sumpter E. (1986). Modification to the compulsive exchange method for measuring exchange characteristics of soils. *Soil Res.* 24 61–66. 10.1071/SR9860061

[B13] Granel T. Robinson B. Mills T. Clothier B. Green S. Fung L. (2002). Cadmium accumulation by willow clones used for soil conservation, stock fodder, and phytoremediation. *Soil Res.* 40 1331–1337. 10.1071/SR02031

[B14] Guadagnini M. (2000). *In vitro-Breeding for Metal-Accumulation in Two Tobacco (Nicotiana tabacum) Cultivars*. Inaugural-Dissertation, Mathematisch Naturwissenschftlichen Fakultät der Universität Freiburg, Breisgau, 109.

[B15] Hammer D. Keller C. (2002). Changes in the rhizosphere of metal-accumulating plants evidenced by chemical extractants. *J. Environ. Qual.* 31 1561–1569. 10.2134/jeq2002.1561 12371173

[B16] Hammer D. Kayser A. Keller C. (2003). Phytoextraction of Cd and Zn with *Salix viminalis* in field trials. *Soil Use Manage.* 19 187–192. 10.1111/j.1475-2743.2003.tb00303.x

[B17] Herzig R. Nehnevajova E. Pfistner C. Schwitzguébel J.-P. Ricci A. Keller C. (2014). Feasibility of labile Zn phytoextraction using enhanced tobacco and sunflower: results of five- and one-year field-scale experiments in Switzerland. *Int. J. Phytoremediat.* 16 735–754. 10.1080/15226514.2013.856846 24933882

[B18] Hogervorst J. Plusquin M. Vangronsveld J. Nawrot T. Cuypers A. Van Hecke E. (2007). House dust as possible route of environmental exposure to cadmium and lead in the adult general population. *Environ. Res.* 103 30–37. 10.1016/j.envres.2006.05.009 16843453

[B19] Jensen J. K. Holm P. E. Nejrup J. Larsen M. B. Borggaard O. K. (2009). The potential of willow for remediation of heavy metal polluted calcareous urban soils. *Environ. Pollut.* 157 931–937. 10.1016/j.envpol.2008.10.024 19062141

[B20] Karlaganis G. (2001). Swiss concept of soil protection, commentary on the ordinance of 1 July 1998 relating to impacts on the soil (OIS). *J. Soils Sediments* 1 239–254. 10.1007/BF02987732

[B21] Kayser A. Wenger K. Keller A. Attinger W. Felix H. Gupta S. (2000). Enhancement of phytoextraction of Zn, Cd, and Cu from calcareous soil: the use of NTA and sulfur amendments. *Environ. Sci. Technol.* 34 1778–1783. 10.1021/es990697s

[B22] Keller C. Hammer D. (2004). Metal availability and soil toxicity after repeated croppings of *Thlaspi caerulescens* in metal contaminated soils. *Environ. Pollut.* 131 243–254. 10.1016/j.envpol.2004.02.030 15234091

[B23] Kennen K. Kirkwood N. (2015). *Phyto: Principles and Resources for Site Remediation and Landscape Design*, 1st Edn. New York, NY: Routledge, 346. 10.4324/9781315746661

[B24] Klang-Westin E. Eriksson J. (2003). Potential of *Salix* as phytoextractor for Cd on moderately contaminated soils. *Plant Soil* 249 127–137. 10.1023/A:1022585404481 28914778

[B25] Koopmans G. Römkens P. Song J. Temminghoff E. Japenga J. (2007). Predicting the phytoextraction duration to remediate heavy metal contaminated soils. *Water Air Soil Pollut.* 181 355–371. 10.1007/s11270-006-9307-7

[B26] Kötschau A. Büchel G. Einax J. W. von Tümpling W. Merten D. (2014). Sunflower (*Helianthus annuus*): phytoextraction capacity for heavy metals on a mining-influenced area in Thuringia, Germany. *Environ. Earth Sci.* 72 2023–2031. 10.1007/s12665-014-3111-2

[B27] Kumpiene J. Bert V. Dimitriou I. Eriksson J. Friesl-Hanl W. Galazka R. (2014). Selecting chemical and ecotoxicological test batteries for risk assessment of trace element-contaminated soils (phyto) managed by gentle remediation options (GRO). *Sci. Total Environ.* 496 510–522. 10.1016/j.scitotenv.2014.06.130 25108253

[B28] Landberg T. Greger M. (1996). Differences in uptake and tolerance to heavy metals in *Salix* from unpolluted and polluted areas. *Appl. Geochem.* 11 175–180. 10.1016/0883-2927(95)00082-8

[B29] Landberg T. Greger M. (2002). Interclonal variation of heavy metal interactions in *Salix viminalis*. *Environ. Toxicol. Chem.* 21 2669–2674. 10.1897/1551-5028(2002)021<2669:IVOHMI>2.0.CO;2 12463563

[B30] Laureysens I. Bogaert J. Blust R. Ceulemans R. (2004a). Biomass production of 17 poplar clones in a short-rotation coppice culture on a waste disposal site and its relation to soil characteristics. *For. Ecol. Manage.* 187 295–309. 10.1016/j.foreco.2003.07.005

[B31] Laureysens I. Blust R. De Temmerman L. Lemmens C. Ceulemans R. (2004b). Clonal variation in heavy metal accumulation and biomass production in a poplar coppice culture: I. Seasonal variation in leaf, wood and bark concentrations. *Environ. Pollut.* 131 485–494. 10.1016/j.envpol.2004.02.009 15261412

[B32] Manzoni S. Molini A. Porporato A. (2011). “Stochastic modelling of phytoremediation,” in *Proceedings of the Royal Society of London A: Mathematical, Physical and Engineering Sciences, rspa20110209*, London 10.1098/rspa.2011.0209

[B33] Maxted A. Black C. West H. Crout N. McGrath S. Young S. (2007). Phytoextraction of cadmium and zinc by *Sailix* from soil historically amended with sewage sludge. *Plant Soil* 290 157–172. 10.1007/s11104-006-9149-5 17379365

[B34] McLaughlin M. J. Zarcinas B. Stevens D. Cook N. (2000). Soil testing for heavy metals. *Commun. Soil Science Plant Anal.* 31 1661–1700. 10.1080/00103620009370531

[B35] Meers E. Du Laing G. Unamuno V. Ruttens A. Vangronsveld J. Tack F. (2007a). Comparison of cadmium extractability from soils by commonly used single extraction protocols. *Geoderma* 141 247–259. 10.1016/j.geoderma.2007.06.002

[B36] Meers E. Samson R. Tack F. Ruttens A. Vandegehuchte M. Vangronsveld J. (2007b). Phytoavailability assessment of heavy metals in soils by single extractions and accumulation by *Phaseolus vulgaris*. *Environ. Exp. Bot.* 60 385–396. 10.1016/j.envexpbot.2006.12.010

[B37] Meers E. Vandecasteele B. Ruttens A. Vangronsveld J. Tack F. (2007c). Potential of five willow species (*Salix* spp.) for phytoextraction of heavy metals. *Environ. Exp. Bot.* 60 57–68. 10.1016/j.envexpbot.2006.06.008

[B38] Mench M. Tancogne J. Gomez A. Juste C. (1989). Cadmium bioavailability to *Nicotiana tabacum* L., *Nicotiana rustica* L. and *Zea mays* L. grown in soil amended or not amended with cadmium nitrate. *Biol. Fertil. Soils*, 8 48–53. 10.1007/BF00260515

[B39] Mench M. Lepp N. Bert V. Schwitzguébel J.-P. Gawronski S. Schröder P. (2010). Successes and limitations of phytotechnologies at field scale: outcomes, assessment and outlook from COST Action 859. *J. Soils Sediments* 10 1039–1070. 10.1007/s11368-010-0190-x

[B40] Mleczek M. Rutkowski P. Rissmann I. Kaczmarek Z. Golinski P. Szentner K. (2010). Biomass productivity and phytoremediation potential of *Salix alba* and *Salix viminalis*. *Biomass Bioenergy* 34 1410–1418. 10.1016/j.biombioe.2010.04.012

[B41] Mola-Yudego B. Díaz-Yáñez O. Dimitriou I. (2015). How much yield should we expect from fast-growing plantations for energy? Divergences between experiments and commercial willow plantations. *BioEnergy Res.* 8 1769–1777. 10.1007/s12155-015-9630-1

[B42] Nehnevajova E. Herzig R. Federer G. Erismann K.-H. Schwitzguébel J.-P. (2007). Chemical mutagenesis—a promising technique to increase metal concentration and extraction in sunflowers. *Int. J. Phytoremediat.* 9 149–165. 10.1080/15226510701232880 18246722

[B43] Nehnevajova E. Herzig R. Bourigault C. Bangerter S. Schwitzguébel J.-P. (2009). Stability of enhanced yield and metal uptake by sunflower mutants for improved phytoremediation. *Int. J. Phytoremediat.* 11 329–346. 10.1080/15226510802565394

[B44] Onwubuya K. Cundy A. Puschenreiter M. Kumpiene J. Bone B. Greaves J. (2009). Developing decision support tools for the selection of “gentle” remediation approaches. *Sci. Total Environ.* 407 6132–6142. 10.1016/j.scitotenv.2009.08.017 19773018

[B45] Perttu K. Kowalik P. (1997). *Salix* vegetation filters for purification of waters and soils. *Biomass Bioenergy* 12 9–19. 10.1016/S0961-9534(96)00063-3

[B46] R Development Core Team (2013). *R: A Language and Environment for Statistical Computing*. Vienna: R Foundation for Statistical Computing Available at: http://www.R-project.org

[B47] Rivelli A. Puschenreiter M. De Maria S. (2014). Assessment of cadmium uptake and nutrient content in sunflower plants grown under Cd stress. *Plant Soil Environ.* 60 80–86. 10.17221/520/2013-PSE

[B48] Robinson B. H. Mills T. M. Petit D. Fung L. E. Green S. R. Clothier B. E. (2000). Natural and induced cadmium-accumulation in poplar and willow: implications for phytoremediation. *Plant Soil* 227 301–306. 10.1023/A:1026515007319

[B49] Robinson B. Fernndez J.-E. Madejón P. Marañón T. Murillo J. M. Green S. (2003). Phytoextraction: an assessment of biogeochemical and economic viability. *Plant Soil* 249 117–125. 10.1023/A:1022586524971

[B50] Robinson B. Schulin R. Nowack B. Roulier S. Menon M. Clothier B. (2006). Phytoremediation for the management of metal flux in contaminated sites. *For. Snow Landsc. Res.* 80 221–224.

[B51] Rosselli W. Keller C. Boschi K. (2003). Phytoextraction capacity of trees growing on a metal contaminated soil. *Plant Soil* 256 265–272. 10.1023/A:1026100707797

[B52] Ruttens A. Boulet J. Weyens N. Smeets K. Adriaensen K. Meers E. (2011). Short rotation coppice culture of willows and poplars as energy crops on metal contaminated agricultural soils. *Int. J. Phytoremediat.* 13 194–207. 10.1080/15226514.2011.568543 22046760

[B53] Vangronsveld J. Van Assche F. Clijsters H. (1995). Reclamation of a bare industrial area contaminated by non-ferrous metals – In situ metal immobilization and revegetation. *Environ. Pollut.* 87 51–59. 10.1016/S0269-7491(99)80007-4 15091607

[B54] Van Nevel L. Mertens J. Oorts K. Verheyen K. (2007). Phytoextraction of metals from soils: how far from practice? *Environ. Pollut.* 150 34–40. 10.1016/j.envpol.2007.05.024 17604889

[B55] Van Ranst E. Verloo M. Demeyer A. Pauwels J. M. (1999). *Manual for the Soil Chemistry and Fertility Laboratory: Analytical Methods for Soils and Plants Equipment, and Management of Consumables*. Ghent: Ghent University, Faculty Agricultural and Applied Biological Sciences, 243.

[B56] Van Slycken S. Witters N. Meers E. Peene A. Michels E. Adriaensen K. (2013a). Safe use of metal-contaminated agricultural land by cultivation of energy maize (*Zea mays*). *Environ. Pollut.* 178 375–380. 10.1016/j.envpol.2013.03.032 23607942

[B57] Van Slycken S. Witters N. Meiresonne L. Meers E. Ruttens A. Van Peteghem P. (2013b). Field evaluation of willow under short rotation coppice for phytomanagement of metal-polluted agricultural soils. *Int. J. Phytoremediat.* 15 677–689. 10.1080/15226514.2012.723070 23819267

[B58] Van Slycken S. Meers E. Tack F. Adriaensen K. Ruttens A. Vangronsveld J. (2015). *Energiegewassen op Landbouwgronden Aangerijkt Met Zware Metalen: Eindverslag*. Gent: Universiteit Gent.

[B59] Vangronsveld J. Herzig R. Weyens N. Boulet J. Adriaensen K. Ruttens A. (2009). Phytoremediation of contaminated soils and groundwater: lessons from the field. *Environ. Sci. Pollut. Res.* 16 765–794. 10.1007/s11356-009-0213-6 19557448

[B65] Vlarebo (2008). *Wetgeving Leefmilieu, Natuur en Energie.* Available at: https://navigator.emis.vito.be/mijn-navigator?woId=23676

[B60] Whiting S. N. Leake J. R. McGrath S. P. Baker A. J. (2001). Assessment of Zn mobilization in the rhizosphere of *Thlaspi caerulescens* by bioassay with non-accumulator plants and soil extraction. *Plant Soil* 237 147–156. 10.1023/A:1013365617841

[B61] Zalewska M. Nogalska A. (2014). Phytoextraction potential of sunflower and white mustard plants in zinc-contaminated soil. *Chilean J. Agric. Res.* 74 485–489. 10.4067/S0718-58392014000400016

[B62] Zárubová P. Hejcman M. Vondráčková S. Mrnka L. Száková J. Tlustoš P. (2015). Distribution of P, K, Ca, Mg, Cd, Cu, Fe, Mn, Pb and Zn in wood and bark age classes of willows and poplars used for phytoextraction on soils contaminated by risk elements. *Environ. Sci. Pollut. Res.* 22 18801–18813. 10.1007/s11356-015-5043-0 26201656

[B63] Zegada-Lizarazu W. Elbersen H. W. Cosentino S. L. Zatta A. Alexopoulou E. Monti A. (2010). Agronomic aspects of future energy crops in Europe. *Biofuels Bioprod. Biorefin.* 4 674–691. 10.1002/bbb.242

[B64] Zhang H. Davison W. Knight B. McGrath S. (1998). In situ measurements of solution concentrations and fluxes of trace metals in soils using DGT. *Environ. Sci. Technol.* 32 704–710. 10.1021/es9704388

