# Supplementary material for: Tobacco, Sunflower and High Biomass SRC Clones Show Potential for Trace Metal Phytoextraction on a Moderately Contaminated Field Site in Belgium
Source: Front Plant Sci. 2018 Dec 21;9:1879. doi: 10.3389/fpls.2018.01879 (PMC6308991; doi:10.3389/fpls.2018.01879)
Supplement: Supplementary file 1 [file Table_1.DOCX]

**Supplementary Table 1: Normal and year specific climate data averaged for the field cultivation period of tobacco and sunflower in the years 2012-2014.** Summary of field preparation and maintenance actions performed in the experimental years.

|  |  | **normal^2^** | **2012** | **2013** | **2014** |
| --- | --- | --- | --- | --- | --- |
| **climate data^1^** | mean T (°C) | 17.5 | 17.3 | 18.2 | 17.3 |
| **(June-August)** | mean max. T (°C) | 22.1 | 21.9 | 22.7 | 21.5 |
|  | mean min. temp. (°C) | 13.2 | 13.0 | 13.7 | 13.4 |
|  | mean rel. air hum (%) | 74.7 | 76.7 | 69.3 | 72.7 |
|  | total rainfall (mm) | 224.6 | 271.3 | 169.2 | 348.2 |
|  | total days of rain (d) | 44 | 51 | 28 | 49 |
|  | total hours of sun (h) | 579 | 534 | 654 | 551 |
|  | mean wind vel (m s^-1^) | 2.9 | 3.2 | 3.2 | 3.0 |
|  | mean wind direction | SW | WSW | NNE | NNE-NNW-SW |
| **field** | weed control before planting |  | / | / | herbicide |
| **preparation** | soil cultivation |  | milling | milling | milling |
|  | fertilization |  | mushroom manure | mushroom manure | mushroom manure |
| **maintenance** | weed control after planting |  | tobacco: mowing | / | / |
|  | irrigation |  | first month | first month | after planting |

^1^Climate data were measured by the Royal Meteorological Institute of Belgium (KMI) (50°48’17’’ N; 4°21’27’’ E. ^2^Mean climatological values for the 30-year period 1981-2010.
